# Supplementary figures and images for: Dengue virus reduces AGPAT1 expression to alter phospholipids and enhance infection in Aedes aegypti
Source: PLoS Pathog. 2019 Dec 9;15(12):e1008199. doi: 10.1371/journal.ppat.1008199 (PMC6922471; doi:10.1371/journal.ppat.1008199)

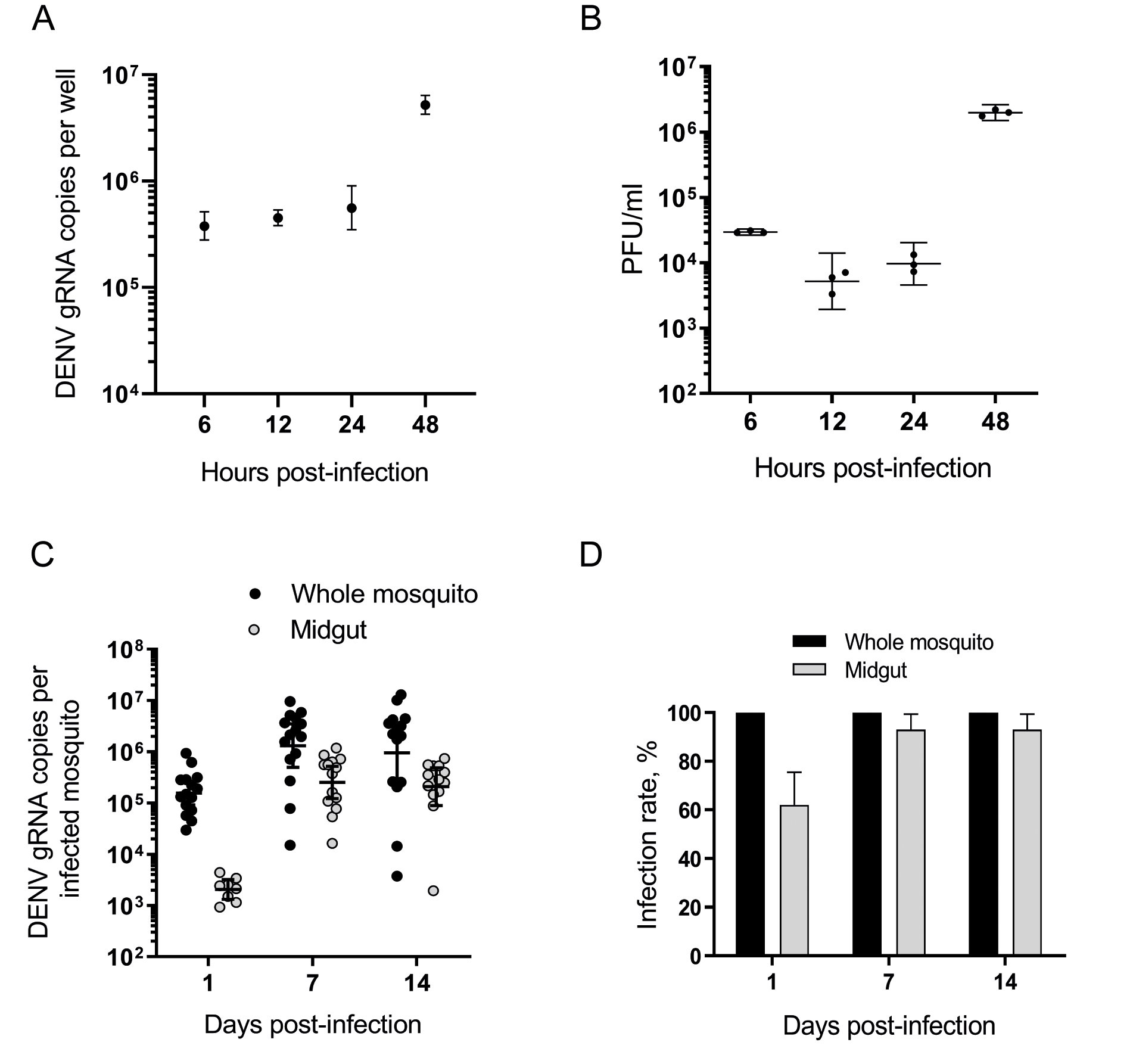

Supplement: S1 Fig — (A) DENV gRNA copies in Aag2 cells at 6, 12, 24 and 48h post-infection with DENV at MOI = 5. Points from 4 repeat and standard errors show geometric mean ± 95% CI. (B) Plaque titer (plaque forming unit—pfu) in supernatant from Aag2 cells at 6, 12, 24 and 48h post-infection. Aag2 cells were infected with DENV at MOI = 5 and virus titer was calculated using plaque assay. Each point represents one well. (C) DENV gRNA copies per infected mosquitoes and dissected midguts at 1, 7 and 14 days post-oral infection with 107 pfu/ml. Each point represents one mosquito or midgut. Bars show geometric mean ± 95% CI. (D) Infection rate in whole mosquito and midgut at 1, 7 and 14 days post-oral infection. Bars represent percentages ± s.e. (TIF) [file ppat.1008199.s001.tif]

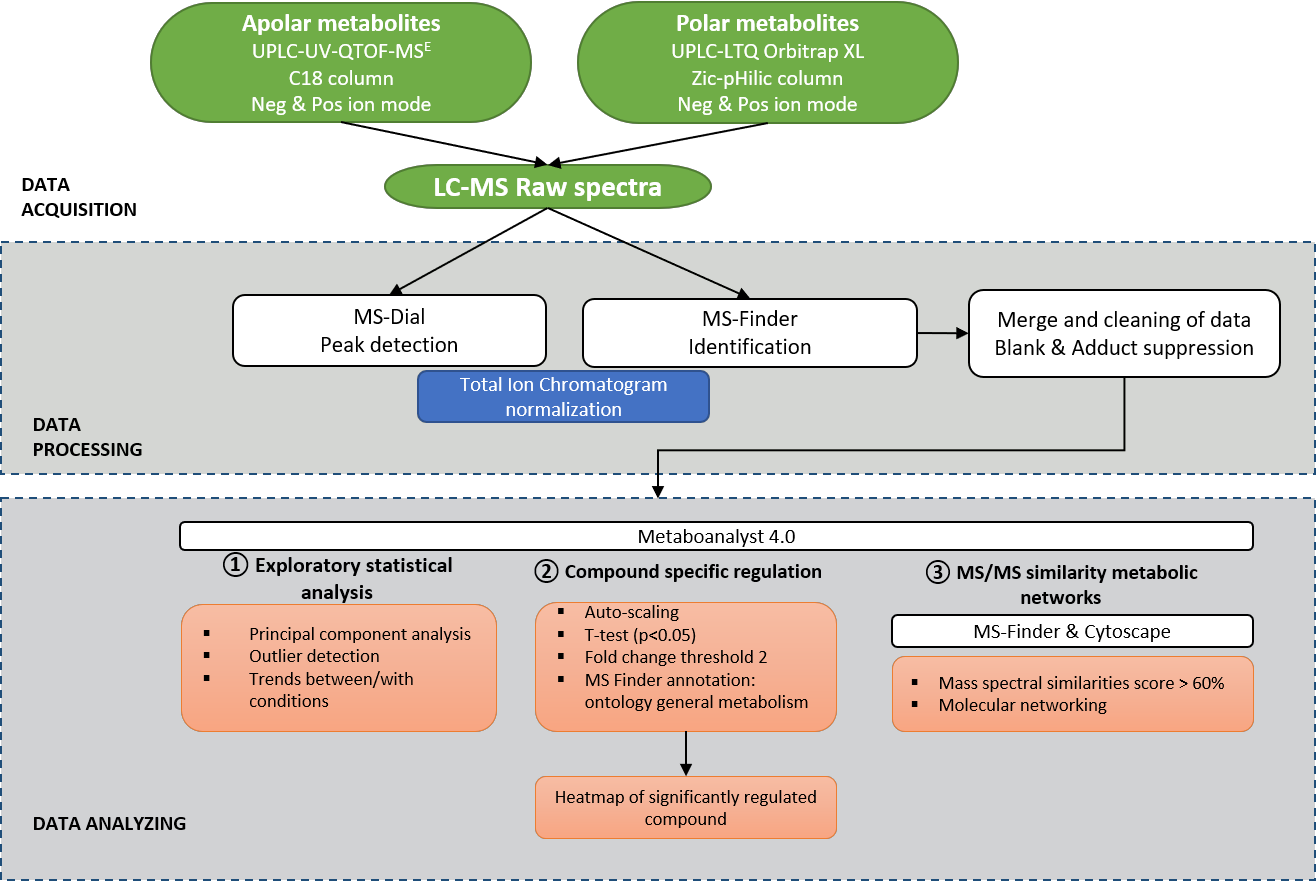

Supplement: S2 Fig — Liquid Chromatography-High Resolution Mass Spectrometry (LC-HRMS) pipeline used to detect polar and nonpolar metabolites. (TIF) [file ppat.1008199.s002.tif]

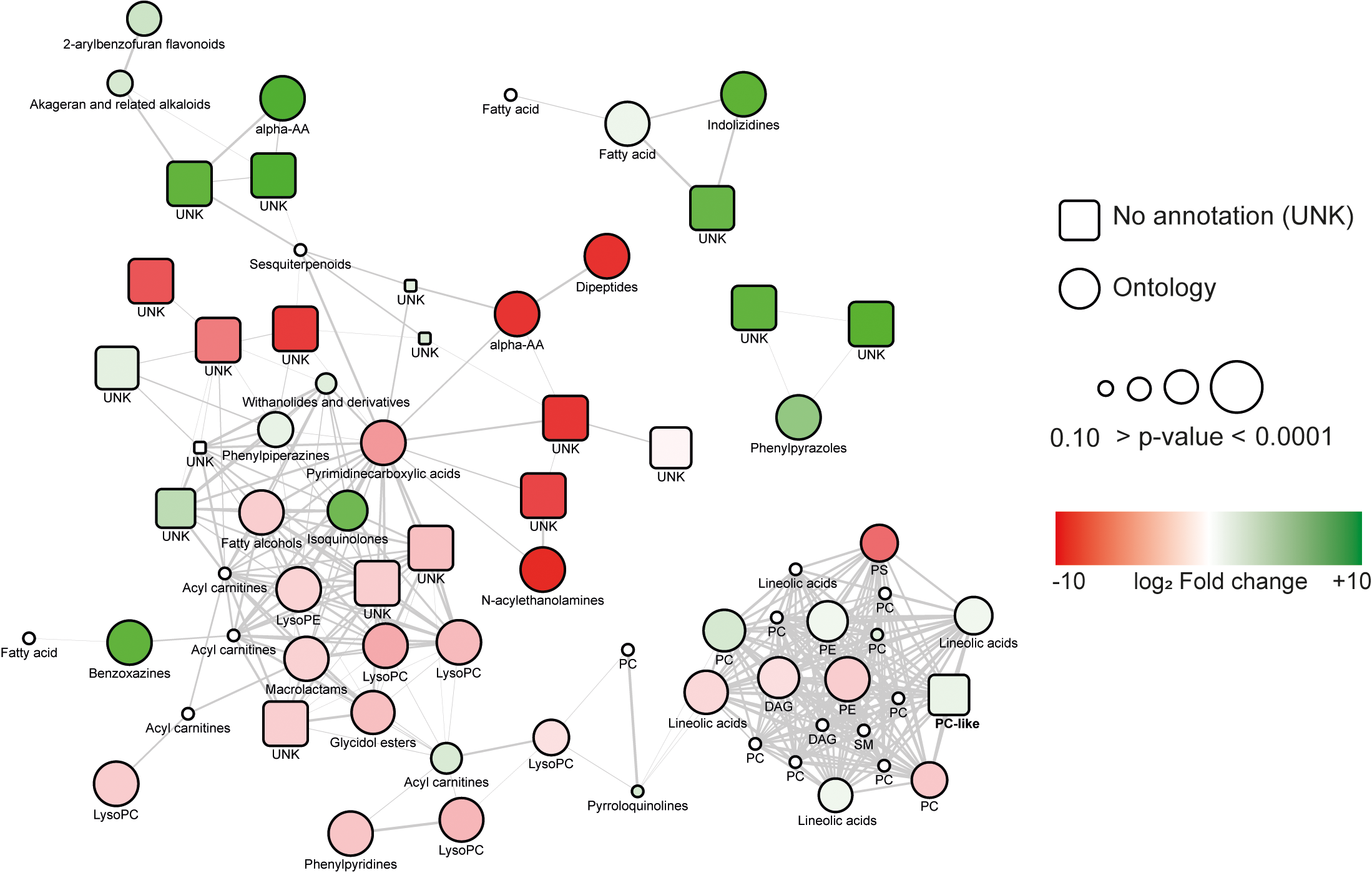

Supplement: S3 Fig — Example of a molecular spectral network for Ae. aegypti mosquito at 14 days post-infection using MS features detected with the non-polar LC condition and MS negative mode. Line length represents the MS/MS score similarity. Ontology for unknown features was determined based on the proximity with database-identified features. (TIF) [file ppat.1008199.s003.tif]

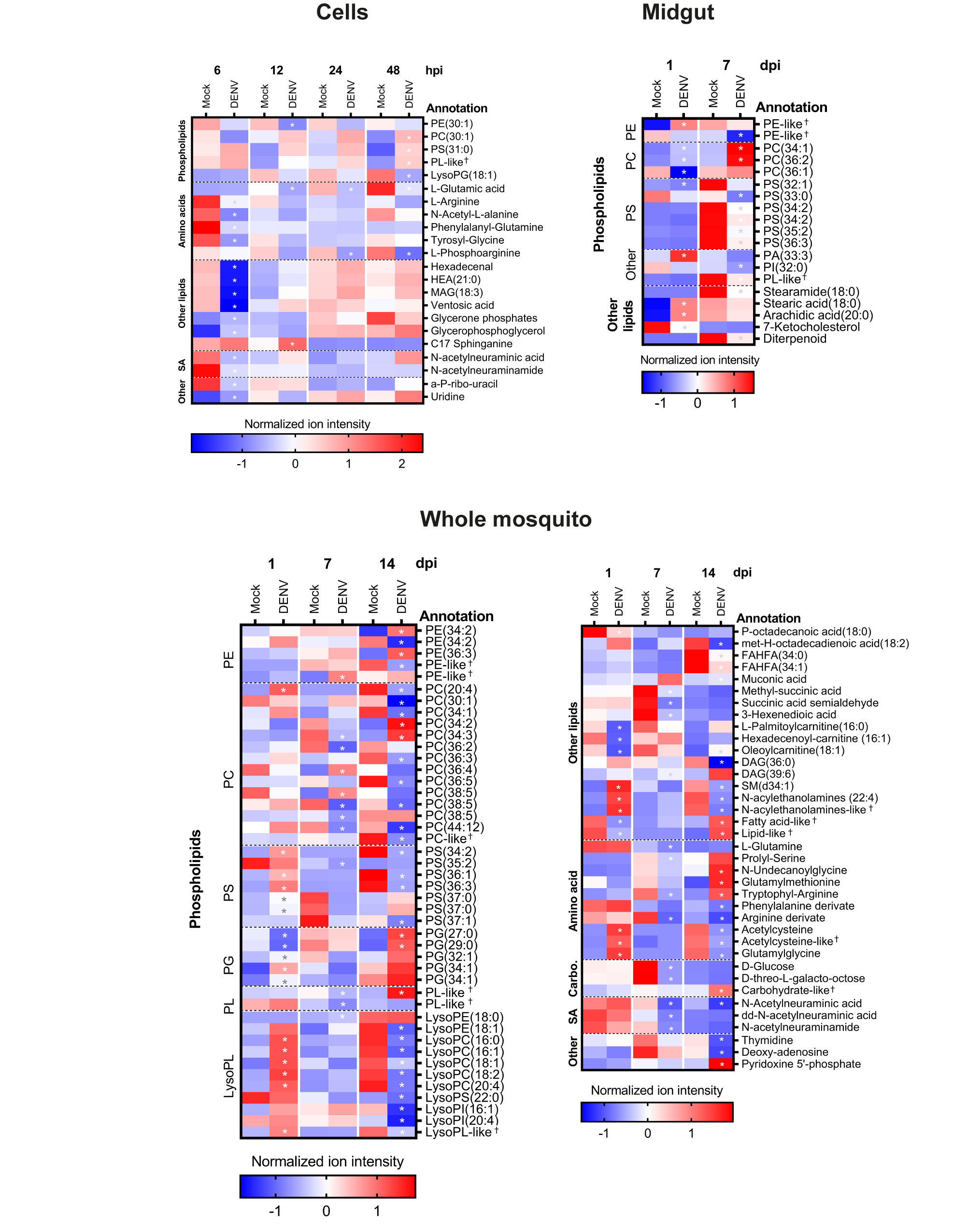

Supplement: S4 Fig — Normalized ion intensity was calculated after total ion chromatography normalization and auto scaling from three biological replicates. Conditions with significantly regulated metabolites (p-value <0.05 and |log2 fold change| >1) were indicated with an asterisk. Only metabolites from the general metabolism (i.e., lipid, carbohydrate, amino acid and peptide, nucleotide and nucleoside, sialic acid) are shown. †, indicates metabolite annotated by spectral similarity. Carbo., carbohydrate; SA, sialic acid; PL, phospholipid; PE, phosphatidylethanolamine; PC, phosphatidylcholine; PS, phosphatidylserine; PA, phosphatidic acid; PI, phosphatidylinositol; PG, phosphatidylglycerol; LysoPS, lysophosphatidylserine; LysoPC, lysophosphatidylcholine; LysoPE, lysophosphatidylethanolamine; LysoPG, lysophosphatidylglycerol; LysoPI, lysophosphatidylinositol; SM, Sphingomyelin; DAG, Diacylglycerol; MAG, Monoacylglycerol; FAHFA, Fatty Acid ester of Hydroxyl Fatty Acid; NAE, N-acylethanolamine; HEA, Heneicosanoic acid; pep., peptides. (TIF) [file ppat.1008199.s004.tif]

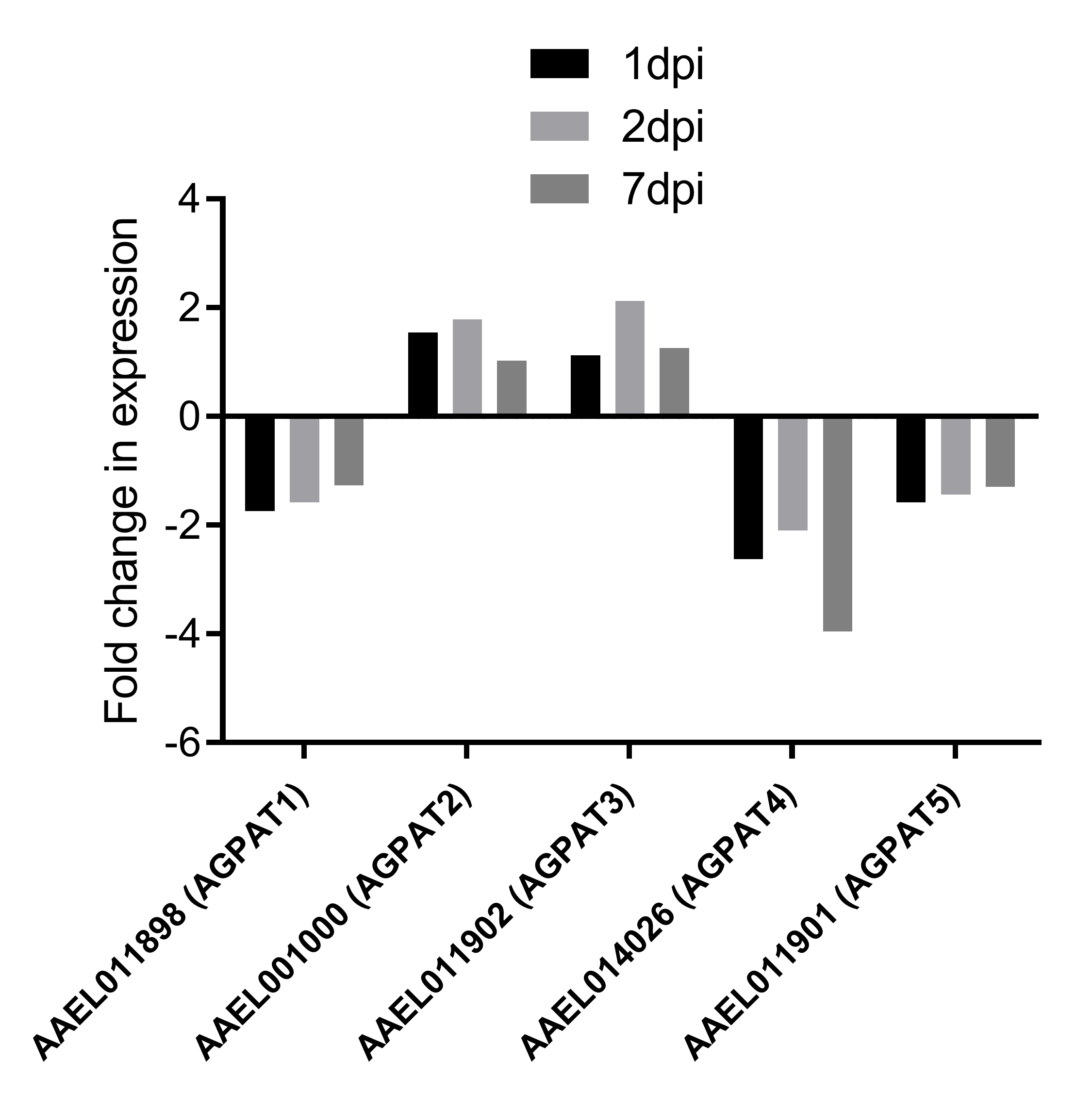

Supplement: S5 Fig — Ae. aegypti female mosquitoes were inoculated with DENV serotype 2 and collected at 1, 2 and 7 days post inoculation for transcriptomic analysis using microarray. Data from 3 separate infections. AGAPAT 1–5 were found significantly regulated by DENV infection. Data retrieved from (Colpitts et al., 2011, PMID: 21909258). (TIF) [file ppat.1008199.s005.tif]

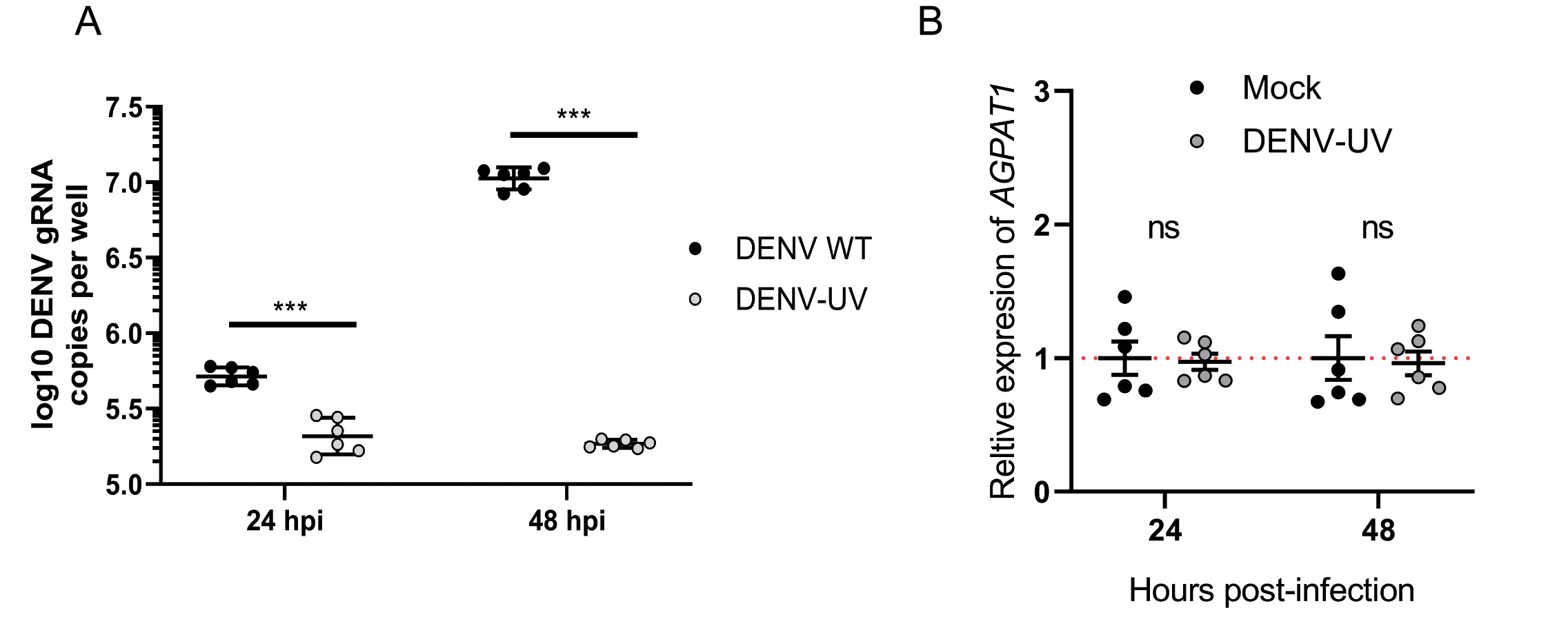

Supplement: S6 Fig — Aag2 cells were infected with an MOI of 5 of DENV (DENV-WT), UV-inactivated DENV (DENV-UV) or mock. Cells were analyzed at 24 and 48 hours post-infection (hpi). (A) DENV gRNA copies. Bars show geometric means ± 95% C.I. (B) AGPAT1 expression relative to Actin level. Bars show arithmetic means ± s.e.m. (A-B) Each point represents an independent well. ***, p-value < 0.001 as determined by unpaired t-test. (TIF) [file ppat.1008199.s006.tif]

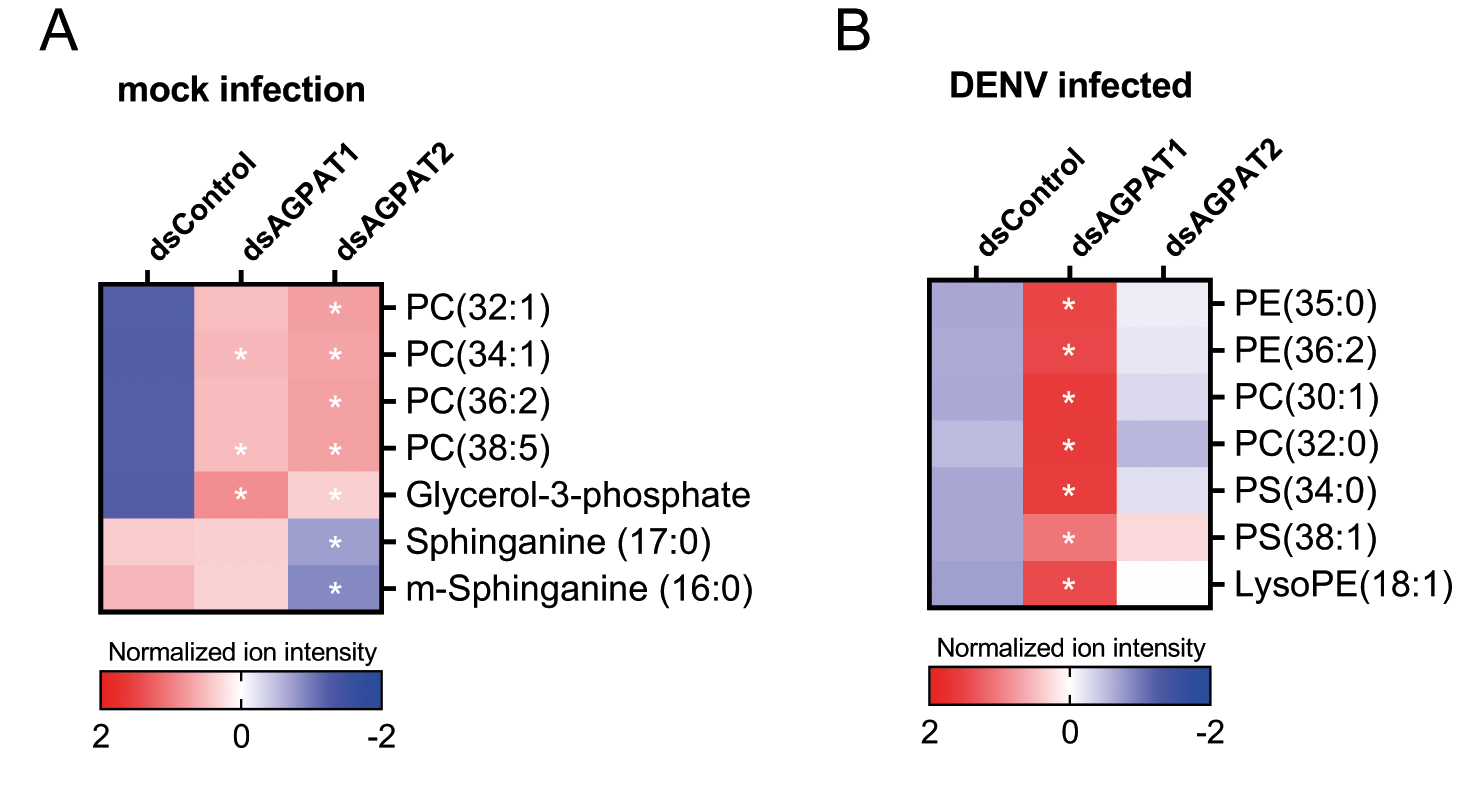

Supplement: S7 Fig — Aag2 cells were transfected with dsRNA against AGPAT1 (dsAGPAT1) or AGPAT2 (dsAGPAT2) or a dsRNA control (dsControl). 24h later, cells were infected with DENV at MOI of 1. (A) Ion intensity of regulated metabolites in mock cells at 72h post transfection. (B) Ion intensity of regulated metabolites in infected cells at 24 hpi. Normalized ion intensity was calculated after total ion chromatography normalization and auto scaling from three replicates. Conditions with significantly regulated metabolites (p-value <0.05 and |log2 fold change| >1) were indicated with an asterisk. PE, phosphatidylethanolamine; PC, phosphatidylcholine; PS, phosphatidylserine; LPE, lysophosphatidylethanolamine. (TIF) [file ppat.1008199.s007.tif]

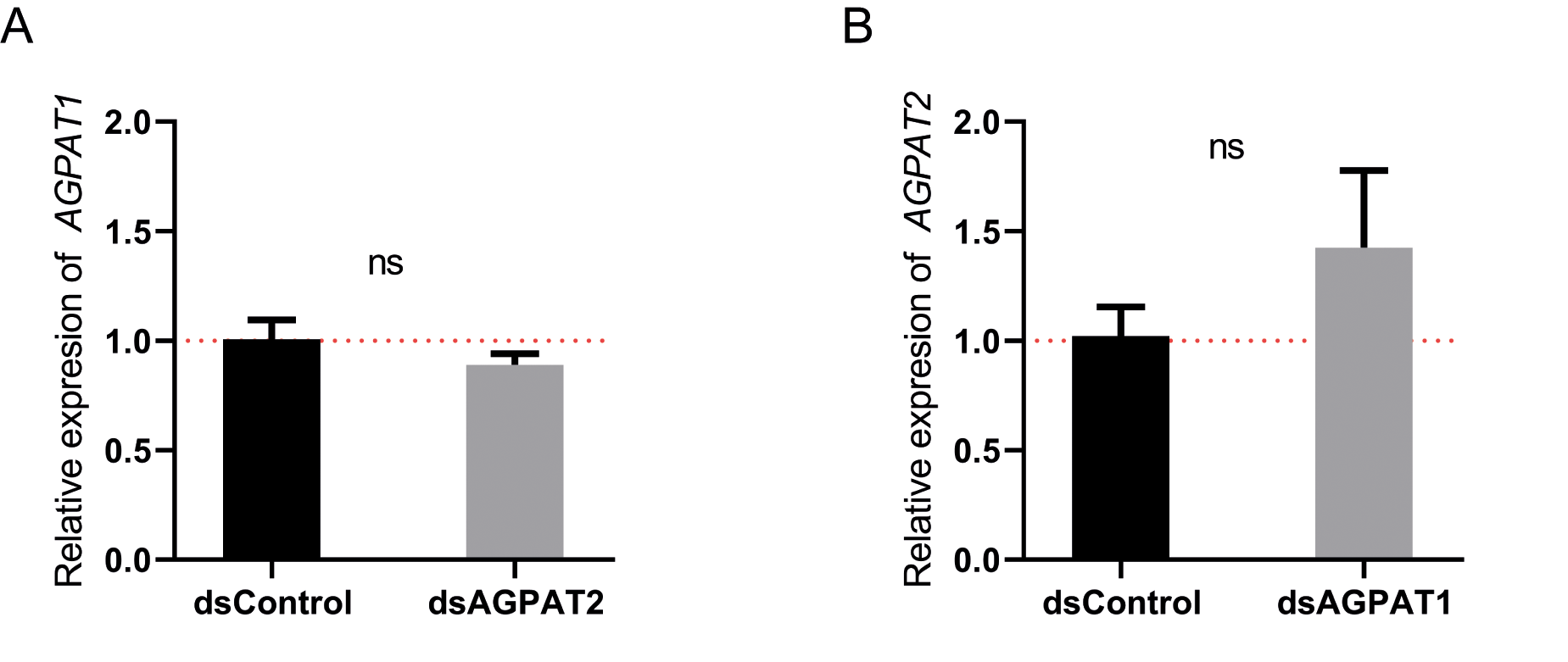

Supplement: S8 Fig — Aag2 cells were transfected with dsRNA against AGPAT1 or 2 (dsAGPAT1 or 2). Control cells were transfected with dsRNA control (dsControl). Cells were collected 72h post dsRNA. (A) AGPAT1 expression in AGPAT2-depleted cells. (B) AGPAT2 expression in AGPAT1-depleted cells. Bars show mean ± s.e.m from 3 biological replicates. ns, non-significant, as indicated by unpaired t-test. (TIF) [file ppat.1008199.s008.tif]

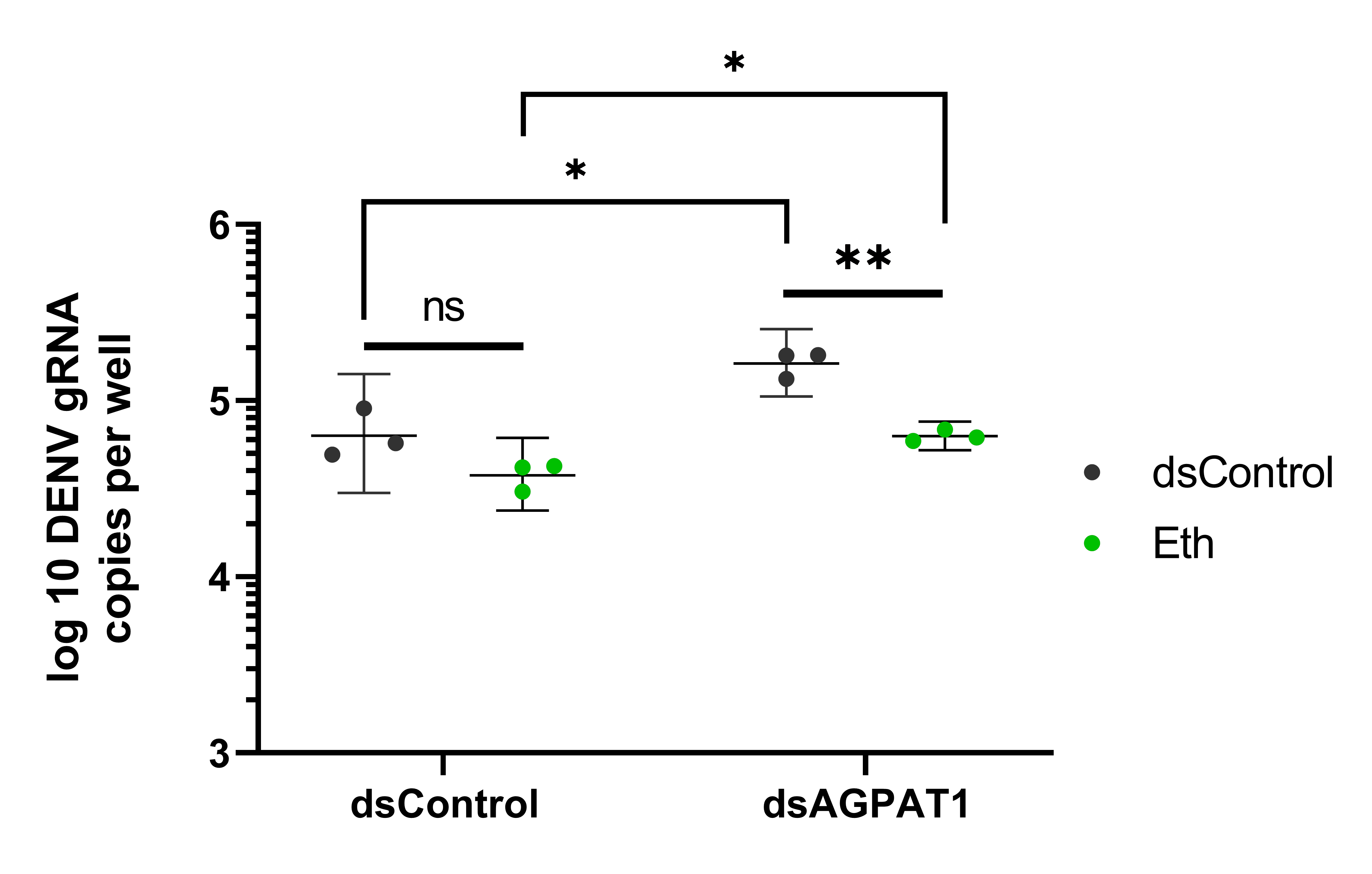

Supplement: S9 Fig — 24h before infection, Aag2 cells were transfected with dsRNA against AGPAT1 (dsAGPAT1) or with dsRNA control (dsControl) and reared in standard growth media or the same media supplemented with 2mM ethanolamine. Cells were infected with DENV at MOI of 1 and gRNA copy was quantified 48h later. Bars show geometric means ± 95% C.I. Each point represents an independent well. *, p-value < 0.05; **, p-value < 0.01 as determined by unpaired t-test. (TIF) [file ppat.1008199.s009.tif]

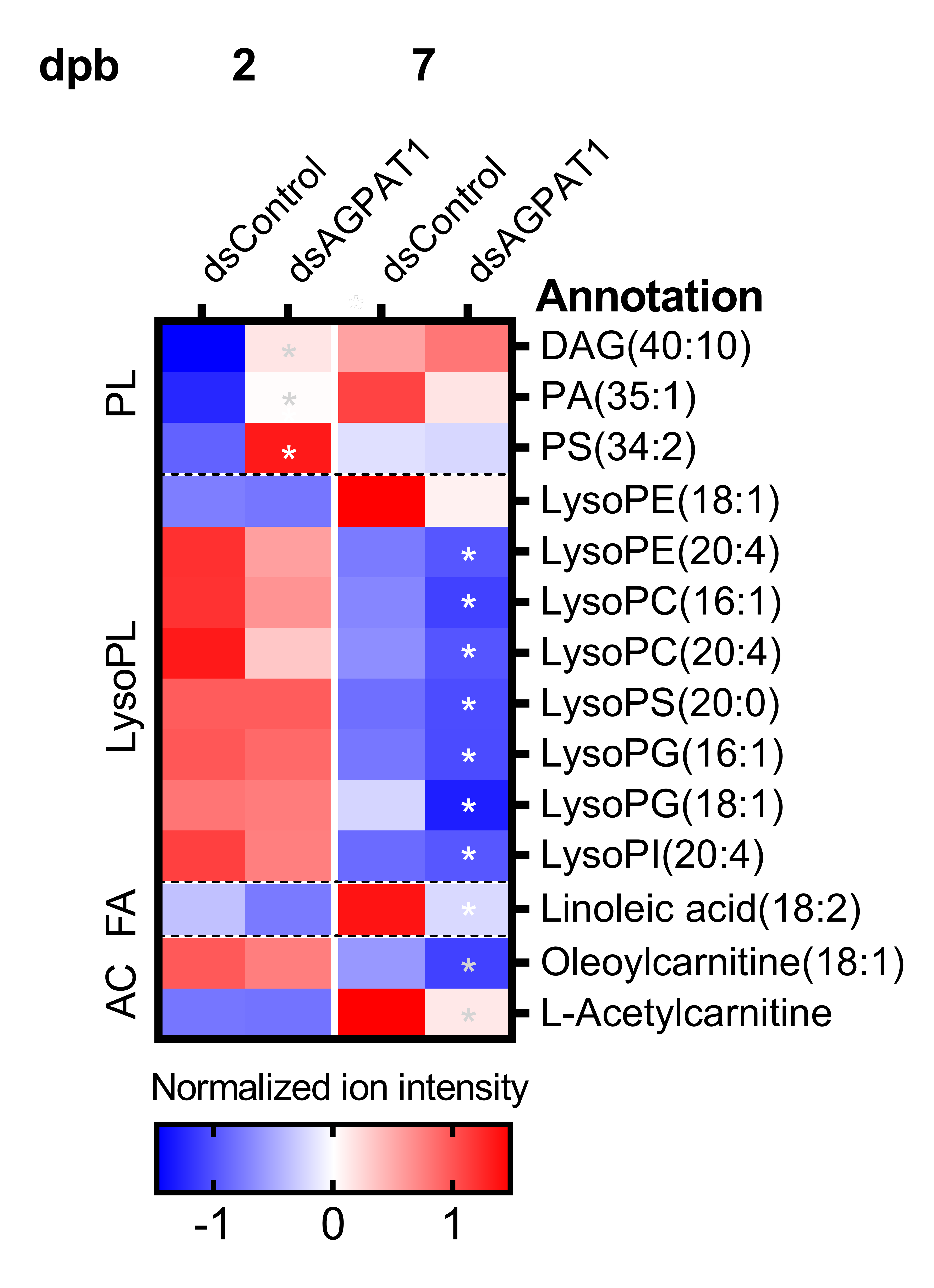

Supplement: S10 Fig — Two days post dsRNA injection against AGPAT1 (dsAGPAT1) or control (dsControl), mosquitoes were orally infected with DENV at 107 pfu/ml. Metabolomic analyses were performed at 2 and 7 dpi. Normalized ion intensity was calculated after total ion chromatography normalization and auto scaling from three replicates. Conditions with significantly regulated metabolites (p-value <0.05 and |log2 fold change| >1) were indicated with an asterisk. (TIF) [file ppat.1008199.s010.tif]

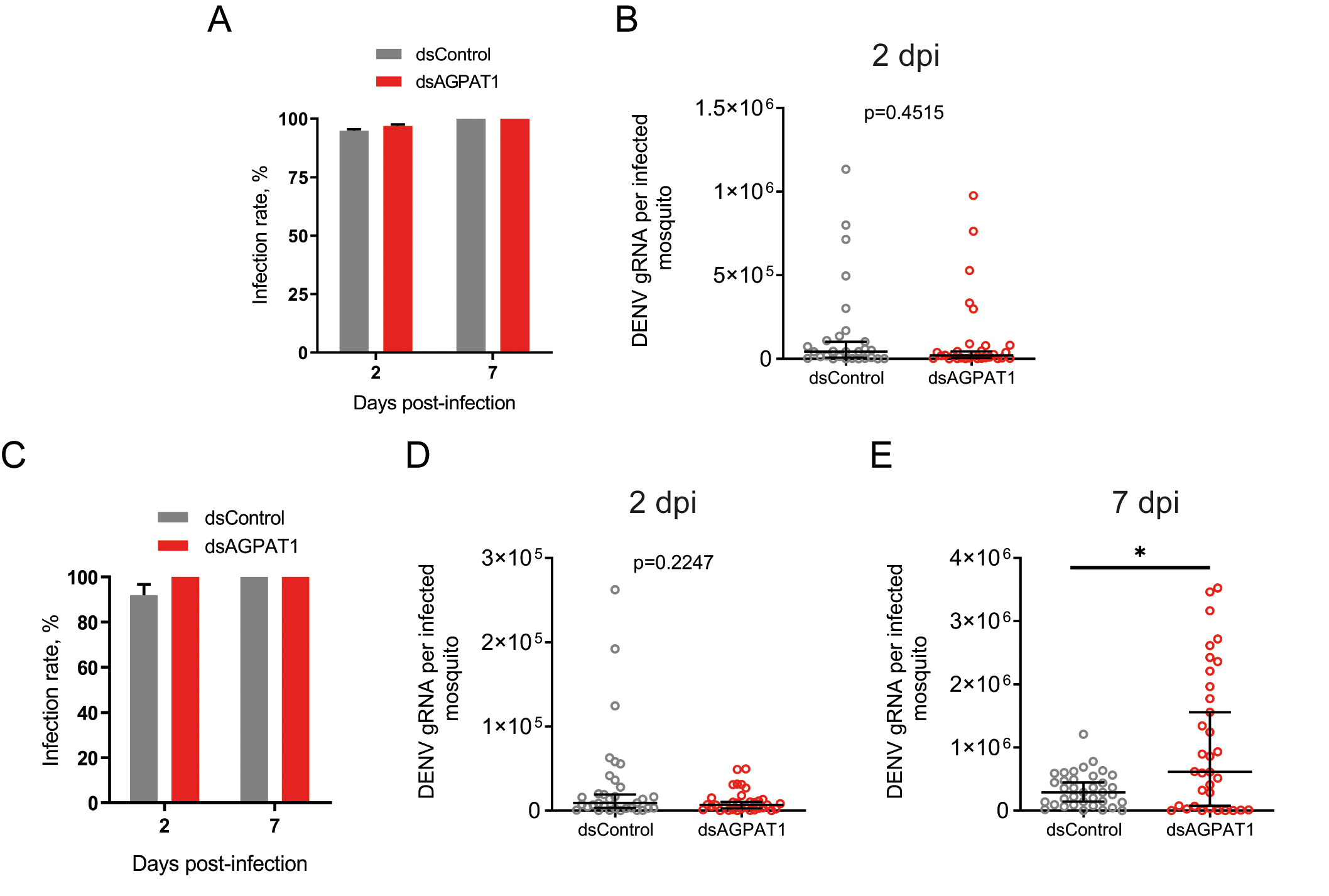

Supplement: S11 Fig — Mosquitoes were injected with either dsRNA against AGPAT1 (dsAGPAT1) or dsRNA control (dsControl). Two days post injection, mosquitoes were orally fed with either non-infectious blood or DENV infectious blood. Impact of AGPAT1 depletion on (A) infection rate and (B) DENV gRNA copies at 2 days post oral infection (dpi) with 107 pfu/ml. (C-E) Impact of AGPAT1 depletion on infection rate (C) and DENV gRNA copies at 2 (D) and 7 (E) dpi with 106 pfu/ml. Bars indicate percentage ± s.e. (A, C) or geometric means ± 95% C.I. (B, D, E) with each dot representing one mosquito. *, p-value < 0.05 as indicated by Mann-Whitney test. (TIF) [file ppat.1008199.s011.tif]
