# Supplementary material for: Dengue virus reduces AGPAT1 expression to alter phospholipids and enhance infection in Aedes aegypti
Source: PLoS Pathog. 2019 Dec 9;15(12):e1008199. doi: 10.1371/journal.ppat.1008199 (PMC6922471; doi:10.1371/journal.ppat.1008199)
Supplement: S1 Table — (DOCX) [file ppat.1008199.s012.docx]

## **Table S1. Identification of mosquito specific metabolites by spectral similarity.**

|  | **Suggested annotation** | **Mass m/z** | **Time course** | **Similarity score** | **Similarity features mass** | **Similarity features Ontology** |
| --- | --- | --- | --- | --- | --- | --- |
| **Cells** | Phospholipid | 1004.0799 | **48 hpi** | 0.67 | 227.2018 | Long-chain fatty acids |
|  |  |  |  | 0.64 | 171.0119 | Glycerophosphates |
| **Midgut** | Phospholipid | 695.311 | **1 dpi** | 0.97 | 716.4861 | Phosphatidylserines |
|  |  |  |  | 0.96 | 742.5027 | Phosphatidylserines |
|  |  |  |  | 0.95 | 714.5056 | Phosphatidylethanolamines |
|  |  |  |  | 0.90 | 740.5221 | Phosphatidylethanolamines |
|  |  |  |  | 0.89 | 711.2864 | Saccharolipids |
|  |  |  |  | 0.88 | 729.4831 | Xanthophylls |
|  |  |  |  | 0.81 | 715.5099 | Acyclic diterpenoids |
|  |  |  |  | 0.80 | 716.5167 | Phosphatidylethanolamines |
|  |  |  |  | 0.76 | 719.4855 | Phosphatidylglycerols |
|  |  |  |  | 0.71 | 740.5191 | Phosphatidylethanolamines |
|  |  |  |  | 0.67 | 717.4706 | Phosphatidylglycerols |
|  | Phospholipid | 701.4858 | **7 dpi** | 0.83 | 711.2864 | Saccharolipids |
|  |  |  |  | 0.81 | 716.5167 | Phosphatidylethanolamines |
|  |  |  |  | 0.78 | 715.5099 | Acyclic diterpenoids |
|  |  |  |  | 0.78 | 729.4831 | Xanthophylls |
|  |  |  |  | 0.74 | 253.2168 | Long-chain fatty acids |
|  |  |  |  | 0.68 | 740.5221 | Phosphatidylethanolamines |
|  |  |  |  | 0.68 | 716.4861 | Phosphatidylserines |
|  |  |  |  | 0.62 | 717.4706 | Phosphatidylglycerols |
|  | Phospholipid | 707.1689 | **7 dpi** | 0.97 | 219.175 | Sesquiterpenoids |
|  |  |  |  | 0.95 | 469.3869 | Stilbenes |
|  |  |  |  | 0.94 | 284.2951 | Carboximidic acids |
|  |  |  |  | 0.93 | 481.3505 | Brassinolides and derivatives |
|  |  |  |  | 0.87 | 553.2563 | Benzodioxoles |
|  |  |  |  | 0.85 | 775.5345 | Glycosyldiacylglycerols |
|  |  |  |  | 0.85 | 850.5543 | Phosphatidylserines |
|  |  |  |  | 0.76 | 437.1938 | 3'-prenylated isoflavanones |
|  |  |  |  | 0.66 | 522.3557 | Lysophosphatidylcholines |
|  |  |  |  | 0.66 | 427.3893 | Cycloartanols and derivatives |
| **Mosquito** | Phospholipid | 846.5413 | **7 dpi** | 0.93 | 794.5101 | Glycosphingolipids |
|  |  |  |  | 0.94 | 818.5106 | Phosphatidylethanolamines |
|  |  |  |  | 0.98 | 820.5248 | Oligopeptides |
|  | Phospholipid | 628.3821 | **14 dpi** | 0.84 | 466.3301 | Lysophosphatidylethanolamines |
|  | Phospholipid | 754.5367 | **14 dpi** | 1.00 | 312.3264 | Morpholines |
|  |  |  |  | 0.99 | 516.307 | Lysophosphatidylcholines |
|  |  |  |  | 0.99 | 758.5696 | Phosphatidylcholines |
|  |  |  |  | 0.91 | 778.5354 | Phosphatidylcholines |
|  |  |  |  | 0.91 | 802.5355 | Phosphatidylcholines |
|  |  |  |  | 0.73 | 808.5806 | Phosphatidylcholines |
|  |  |  |  | 0.69 | 566.323 | Benzoic acids |
|  |  |  |  | 0.67 | 522.356 | Lysophosphatidylcholines |
|  |  |  |  | 0.67 | 284.2961 | Carboximidic acids |
|  |  |  |  | 0.66 | 808.5842 | Phosphatidylcholines |
|  |  |  |  | 0.66 | 780.5518 | Phosphatidylcholines |
|  | Phospholipid | 611.3975 | **7 dpi** | 0.67 | 281.2477 | Long-chain fatty acids |
|  |  |  |  | 0.67 | 659.5121 | Phosphoethanolamines |
|  |  |  |  | 0.65 | 564.3434 | Resorcinols |
|  |  |  |  | 0.64 | 687.4951 | Phosphatidic acid |
|  |  |  |  | 0.64 | 714.4923 | Phosphatidylethanolamines |
|  |  |  |  | 0.63 | 775.5479 | Phosphatidylglycerols |
|  |  |  |  | 0.60 | 734.533 | Phosphatidylserines |
|  | Phospholipid | 768.4946 | **7 / 14 dpi** | 0.97 | 469.2935 | Withanolides and derivatives |
|  |  |  |  | 0.95 | 428.3731 | Acyl carnitines |
|  |  |  |  | 0.90 | 359.1749 | Phenylpiperazines |
|  |  |  |  | 0.89 | 311.2584 | Fatty alcohols |
|  |  |  |  | 0.88 | 518.2646 | Harmala alkaloids |
|  |  |  |  | 0.87 | 453.1676 | Sulfated steroids |
|  |  |  |  | 0.76 | 480.3088 | Lysophosphatidylethanolamines |
|  |  |  |  | 0.61 | 463.2568 | Fatty acyl glycosides |
|  | Lysophospholipid  / Acylcarnitine | 534.2963 | **1 / 14 dpi** | 0.95 | 630.4976 | Aralkylamines |
|  |  |  |  | 0.99 | 339.2893 | Glycidol esters |
|  |  |  |  | 0.98 | 398.3265 | Acyl carnitines |
|  |  |  |  | 0.66 | 426.3578 | Acyl carnitines |
|  |  |  |  | 0.97 | 428.3731 | Acyl carnitines |
|  |  |  |  | 0.61 | 452.277 | Acyl carnitines |
|  |  |  |  | 0.62 | 480.3088 | Lysophosphatidylethanolamines |
|  |  |  |  | 0.98 | 518.2646 | Harmala alkaloids |
|  |  |  |  | 0.64 | 520.3402 | Lysophosphatidylcholines |
|  | Lysophospholipid | 534.2963 | **1 / 14 dpi** | 0.64 | 544.3403 | Lysophosphatidylcholines |
|  |  |  |  | 0.64 | 558.2961 | Diphenylethers |
|  |  |  |  | 0.94 | 560.312 | Macrolactams |
|  | N-acylethanolamines | 376.2597 | **1 / 14 dpi** | 0.83 | 376.3156 | N-acylethanolamines |
|  | Lipid | 107.086 | **1 / 14 dpi** | 0.87 | 219.1751 | Sesquiterpenoids |
|  |  |  |  | 0.72 | 337.1053 | Coumestans |
|  |  |  |  | 0.95 | 135.0809 | Alkylthiols |
|  |  |  |  | 0.90 | 425.1364 | Ginkgolides and bilobalides |
|  | Fatty acid | 192.16 | **1 / 14 dpi** | 0.96 | 216.1964 | Medium-chain fatty acids |
|  |  |  |  | 0.94 | 156.1386 | Indolizidines |
|  | Carbohydrate | 198.1861 | **14 dpi** | 0.69 | 839.3719 | Sugar acids and derivatives |
|  |  |  |  | 0.80 | 359.1749 | Phenylpiperazines |
|  | Acyl-Amino acid | 616.1778 | **7 / 14 dpi** | 0.64 | 164.0408 | N-acyl-L-alpha-amino acids |
|  | Pyrazinecarboxamides | 789.4676 | **1 / 14 dpi** | 0.66 | 414.2152 | Pyrazinecarboxamides |
|  | Hydroxypyrimidines | 234.9183 | **14 dpi** | 0.75 | 184.0742 | Hydroxypyrimidines |
|  | Hydroxypyrimidines | 376.2597 | **1 / 14 dpi** | 0.78 | 184.0739 | Hydroxypyrimidines |
|  |  |  |  | 0.80 | 184.0742 | Hydroxypyrimidines |
|  | Hydroxypyrimidines | 768.4946 | **7 / 14 dpi** | 0.94 | 184.0739 | Hydroxypyrimidines |
|  |  |  |  | 0.97 | 184.0742 | Hydroxypyrimidines |
