# Supplementary material for: Dengue virus reduces AGPAT1 expression to alter phospholipids and enhance infection in Aedes aegypti
Source: PLoS Pathog. 2019 Dec 9;15(12):e1008199. doi: 10.1371/journal.ppat.1008199 (PMC6922471; doi:10.1371/journal.ppat.1008199)
Supplement: S3 Table — (DOCX) [file ppat.1008199.s014.docx]

**Table S3. Primers for dsRNA.**

| Gene name | Gene code | Fragment size | Forward primer | Reverse primer |
| --- | --- | --- | --- | --- |
| *AGPAT1* | AAEL011898 | 364 | taatacgactcactatagggTTAAGCGCATGCCGTAAAAA | taatacgactcactatagggTTGGTACAGAGATAGGCGGG |
| *AGPAT2* | AAEL001000 | 346 | taatacgactcactatagggGGCCTACTTTTGCAGTTTGAA | taatacgactcactatagggCGAGTTGATCATCAGCACAAA |
| *LacZ* | / | 370 | TAATACGACTCACTATAGGGTACCCGTAGGTAGTCACGCA | TAATACGACTCACTATAGGGTACGATGCGCCCATCTACAC |
