# Supplementary material for: Dengue virus reduces AGPAT1 expression to alter phospholipids and enhance infection in Aedes aegypti
Source: PLoS Pathog. 2019 Dec 9;15(12):e1008199. doi: 10.1371/journal.ppat.1008199 (PMC6922471; doi:10.1371/journal.ppat.1008199)
Supplement: S4 Table — (DOCX) [file ppat.1008199.s015.docx]

**Table S4. Primers for Real-Time qPCR**

| Gene name | Gene code | Forward primer | Reverse primer |
| --- | --- | --- | --- |
| *AGPAT1* | AAEL011898 | TAAGCGCATGCCGTAAAAAT | GTGGCCGTAAAAGCATGAG |
| *AGPAT2* | AAEL001000 | GGCCTACTTTTGCAGTTTGAA | CGAGTTGATCATCAGCACAAA |
| *Actin* | AAEL011197 | GAACACCCAGTCCTGCTGACA | TGCGTCATCTTCTCACGGTTAG |
