# Supplementary material for: Dengue virus reduces AGPAT1 expression to alter phospholipids and enhance infection in Aedes aegypti
Source: PLoS Pathog. 2019 Dec 9;15(12):e1008199. doi: 10.1371/journal.ppat.1008199 (PMC6922471; doi:10.1371/journal.ppat.1008199)
Supplement: S5 Table — (DOCX) [file ppat.1008199.s016.docx]

**Table S5. AGPAT FASTA protein sequence.**

>NP_006402.1 1-acyl-sn-glycerol-3-phosphate acyltransferase alpha [Homo sapiens]

MDLWPGAWMLLLLLFLLLLFLLPTLWFCSPSAKYFFKMAFYNGWILFLAVLAIPVCAVRGRNVENMKILR

LMLLHIKYLYGIRVEVRGAHHFPPSQPYVVVSNHQSSLDLLGMMEVLPGRCVPIAKRELLWAGSAGLACW

LAGVIFIDRKRTGDAISVMSEVAQTLLTQDVRVWVFPEGTRNHNGSMLPFKRGAFHLAVQAQVPIVPIVM

SSYQDFYCKKERRFTSGQCQVRVLPPVPTEGLTPDDVPALADRVRHSMLTVFREISTDGRGGGDYLKKPG

GGG

>NP_006403.2 1-acyl-sn-glycerol-3-phosphate acyltransferase beta isoform a precursor [Homo sapiens]

MELWPCLAAALLLLLLLVQLSRAAEFYAKVALYCALCFTVSAVASLVCLLRHGGRTVENMSIIGWFVRSF

KYFYGLRFEVRDPRRLQEARPCVIVSNHQSILDMMGLMEVLPERCVQIAKRELLFLGPVGLIMYLGGVFF

INRQRSSTAMTVMADLGERMVRENLKVWIYPEGTRNDNGDLLPFKKGAFYLAVQAQVPIVPVVYSSFSSF

YNTKKKFFTSGTVTVQVLEAIPTSGLTAADVPALVDTCHRAMRTTFLHISKTPQENGATAGSGVQPAQ

>NP_064517.1 1-acyl-sn-glycerol-3-phosphate acyltransferase gamma [Homo sapiens]

MGLLAFLKTQFVLHLLVGFVFVVSGLVINFVQLCTLALWPVSKQLYRRLNCRLAYSLWSQLVMLLEWWSC

TECTLFTDQATVERFGKEHAVIILNHNFEIDFLCGWTMCERFGVLGSSKVLAKKELLYVPLIGWTWYFLE

IVFCKRKWEEDRDTVVEGLRRLSDYPEYMWFLLYCEGTRFTETKHRVSMEVAAAKGLPVLKYHLLPRTKG

FTTAVKCLRGTVAAVYDVTLNFRGNKNPSLLGILYGKKYEADMCVRRFPLEDIPLDEKEAAQWLHKLYQE

KDALQEIYNQKGMFPGEQFKPARRPWTLLNFLSWATILLSPLFSFVLGVFASGSPLLILTFLGFVGAASF

GVRRLIGVTEIEKGSSYGNQEFKKKE

>NP_064518.1 1-acyl-sn-glycerol-3-phosphate acyltransferase delta [Homo sapiens]

MDLAGLLKSQFLCHLVFCYVFIASGLIINTIQLFTLLLWPINKQLFRKINCRLSYCISSQLVMLLEWWSG

TECTIFTDPRAYLKYGKENAIVVLNHKFEIDFLCGWSLSERFGLLGGSKVLAKKELAYVPIIGWMWYFTE

MVFCSRKWEQDRKTVATSLQHLRDYPEKYFFLIHCEGTRFTEKKHEISMQVARAKGLPRLKHHLLPRTKG

FAITVRSLRNVVSAVYDCTLNFRNNENPTLLGVLNGKKYHADLYVRRIPLEDIPEDDDECSAWLHKLYQE

KDAFQEEYYRTGTFPETPMVPPRRPWTLVNWLFWASLVLYPFFQFLVSMIRSGSSLTLASFILVFFVASV

GVRWMIGVTEIDKGSAYGNSDSKQKLND

>NP_060831.2 1-acyl-sn-glycerol-3-phosphate acyltransferase epsilon [Homo sapiens]

MLLSLVLHTYSMRYLLPSVVLLGTAPTYVLAWGVWRLLSAFLPARFYQALDDRLYCVYQSMVLFFFENYT

GVQILLYGDLPKNKENIIYLANHQSTVDWIVADILAIRQNALGHVRYVLKEGLKWLPLYGCYFAQHGGIY

VKRSAKFNEKEMRNKLQSYVDAGTPMYLVIFPEGTRYNPEQTKVLSASQAFAAQRGLAVLKHVLTPRIKA

THVAFDCMKNYLDAIYDVTVVYEGKDDGGQRRESPTMTEFLCKECPKIHIHIDRIDKKDVPEEQEHMRRW

LHERFEIKDKMLIEFYESPDPERRKRFPGKSVNSKLSIKKTLPSMLILSGLTAGMLMTDAGRKLYVNTWI

YGTLLGCLWVTIKA

>EAT35978.1 AAEL011898-PA [Aedes aegypti]

MTTTNSELLGLAFMAFFIITLSSTARYYFKFFCFIILSVVCAVGPVPLMLLRPRDYRNALLPAYLCTKFG

KMLGASFEVRGRENVNRQHGGVVLMNHQSALDLVVLAYLWPIVGRATVVAKREVLYMFPFGLACWLWGTL

FINRKNQRSAKSAINNESKAINEKQAKILFFPEGTRGDGDSLLPFKKGSFHVAIEAQGYIQPVVISKYHF

LNSKAKLFNRGQNIIKILPEVSCVGLTKDDMPQLMDRVQRMMQSEYEALSDESLAINNLSKSL

>EAT47921.1 AAEL001000-PA [Aedes aegypti]

MKAYFCSLKLFSLFPTLRHATWPHFLFLQRDDISAAITSEPTSSCDDRVLLFYFQNVGGMNTTLAKYLLA

CKGTMASYYEIFLICGIILMPIFYETSHKFRYFFKFFIYYFVLMINSIILIPAMMFRAKDVRNLIWAGTF

CRPISTVLGIKWELRGADILSRDEAYVIVANHQSSLDILGMFDFWHVMNKCTVIAKKELLYTGPFGVAAW

LSGLIFIDRKNAEKAHVAMNECTDMLKEKRIKLWVFPEGTRRNTNEIHPFKKGAFHTAVRSQLPIMPVVY

SSYGSFLDDKAKILNNGHVIVTTLEPIETKGLTSDDIPELMERVRNVMMDTFKATTKEVENKYSVNSTKN

GGVGLSGSKLRLRCIDDLIKPKLASSRRLNASANGSPTKESAVYRRKE

>EAT35981.1 AAEL011902-PA [Aedes aegypti]

MTDCTLCHYVGLLVKYYLYAWIIGVGVWFLLIIASKVGSDGNKFRYYAKYGMIYYATQAFTTLFAPFSLL

RPRNPANAGIICAVASKASSLLPITWELRNARILREAEGAVVMANHQSSMDILGLEILWSTMRNVISIAK

KEMLFIVPFGPAAWLAGITFINRKNRPSAMKTLDGCKRKMVEQGFKMYIYPEGTRFPERGMLPFKKGGFH

TAIEAGVPIIPVVFSHIYFIDAKKYSFKPGHVIMNVLEPIPTKGLTKDNLDALITRTRDAMMAEYERLSA

EMDANLANPKWVKASRPRFVTYDGKKTN

>EAT33698.1 AAEL014026-PA [Aedes aegypti]

MHLCFAISYFTSGLIINTAQCILYFGLKPFNKRLYRKIGYYLCYSFYSQLVFLADWWSGSTLYIYISDED

LKHCGKEHVLLLMNHTYEVDWLVGWMFCEKVKVLGNCKAYAKKVIQYIPTVGWAWKFAEFVFLERSFDKD

KEIIGRQIKEIMDYPDPVWLLLNAEGTRFTEKKHEASIKFARDRGMVELKHHLIPRTKGFTASLPELRNK

STILDIQLAISKDSPVKPTIFNILNGKPIEAHMHIRRIPFDQVPEDEGQAAEWLQELFRQKDVMQESFHK

HGDFFTGSNVTRKVPVKLHPRLHTLINMVAWNVLTVVPMFYYLIQLLISGEIMYFSIGTSILIAFYGLMV

KAIGMSKISKASSYGSEKKNGQSVHNGPSSNETTKNK

>EAT35980.1 AAEL011901-PA [Aedes aegypti]

MAMEAIISTIKDVFLGSTCVQIMVVSILLSLVWPTFKYYAKLTAILMMSFMVMVVPIPLYFFKPRWPLNA

LIPGIVACEIIRWFGVEYEIRGKENINVKNGGVALINHQSAIDIVMLSRLLREFRNIVPVVKKELFYALP

FGIASYLVGVVFIDRKNITSAKDVMKREAVAIQRDNLKLAIFPEGTRHDKDTLLPFKKGSFHVAIDSQSI

IQSIIVSKYGFLDHKKKRFGRGRVIIKILPEISTKGMTKDDINSLVEKCQTTMQAEFDALSAEAKQYCHL
