# Supplementary material for: Dengue virus reduces AGPAT1 expression to alter phospholipids and enhance infection in Aedes aegypti
Source: PLoS Pathog. 2019 Dec 9;15(12):e1008199. doi: 10.1371/journal.ppat.1008199 (PMC6922471; doi:10.1371/journal.ppat.1008199)
Supplement: Attachment — Submitted filename: Konan REVIEW-Vial et al PPAT-D-19-00992-070319.docx [file ppat.1008199.s022.docx]

**Vial et al. PPATHOGENS-D-19-00992**

This study aims to understand how dengue virus (DENV) modulates mosquito metabolome to enhance virus production. Using high-resolution mass spectrometry, the authors identified changes in aminophospholipid (aminoPL) concentrations during the course of DENV infection in vitro and in vivo. They showed that the expression of 1-Acylglycerol-3-Phosphate O-Acyltransferase 1 (AGPAT1), a rate-limiting enzyme of phospholipid biogenesis, is decreased during DENV infection, whereas AGPAT1 knockdown leads to a modest increase in DENV production late in infection. This is a significant, but highly descriptive study. There is little mechanism as to how DENV utilizes a select group of phospholipids (PL) to enhance virus production. The study does not address how DENV infection causes AGPTA1 inhibition or the relationship between AGPAT1 inhibition and an increase in aminoPL concentration. It is unclear how in aminoPLs contribute to DENV infectivity in mosquitoes. Finally, if AGPTA1 plays a crucial role in DENV infectivity, its silencing should lead to a marked increase in DENV load. However, this is not the case in the current study.

**Specific comments**

Fig. 1 addresses the impact of DENV infection on Ae. Aegypti metabolome. In Fig. 1B, the Venn diagrams and related triangles need to be explained. In addition, there are no controls for the heat maps in Fig. 1C, making it hard to judge the data.

Fig. 2C-E show a decrease in AGPTA1 mRNA levels, during the course of DENV infection. These data need to be validated with AGPTA1 protein expression during DENV infection.

Fig. 3 addresses the impact of AGPAT silencing on aminoPL levels and DENV replication in mosquito cells. First, it will be helpful to show the control PL samples for the heat maps in Fig. 3C and 3E. It is hard to compare the PL heat maps, from mock and DENV-infected cells, when they show different PL species. In Fig. 3D, it is also hard to interpret the DENV infection data based on the heat maps in Fig. 3C and 3E.There is only a modest increase in DENV production after AGPAT1 knockdown.

Fig. 4 addresses the impact of AGPAT1 silencing on DENV replication and aminoPL consumption in mosquitoes. Please, show the heat map for control siRNA-treated mosquitoes in Fig. 4B. In Fig. 4CD, contrary to the authors assertion, AGPAT1 knockdown in mosquitoes shows no significant impact on DENV infectivity at day 2 or 7 postinfection. The DENV infection rate at day 7 (Fig. 4C) is clearly not impacted by AGPAT1 knockdown. There might be a slight increase in DENV genome replication at day 7, but is there a significant change in DENV load in these mosquitoes?
