# Supplementary material for: Dengue virus reduces AGPAT1 expression to alter phospholipids and enhance infection in Aedes aegypti
Source: PLoS Pathog. 2019 Dec 9;15(12):e1008199. doi: 10.1371/journal.ppat.1008199 (PMC6922471; doi:10.1371/journal.ppat.1008199)
Supplement: Attachment — Submitted filename: PPATHOGENS-D-19-00992 answer to reviewerscomments.docx [file ppat.1008199.s023.docx]

**PPATHOGENS-D-19-00992**

Dear Editor and Reviewers,

We thank the reviewers for their time and comments that helped us improve the manuscript. We added new data to answer these (Fig. 2D, Fig. 3F, Fig. 4D, Fig. S6), modified the text as requested and did our best to satisfy the curiosity on the mechanistic implications of our findings. We are particularly thankful to them for acknowledging the “implications for the field’s understanding of dengue-induced metabolic remodeling” (R1), the significance (R2) and the added mechanistic value compared to previous studies published in PLoS Pathogens (R3). We suspect the R3 refers to Chotiwan et al 2018.

Please find below a point-by-point answer to reviewers’ comments in blue.

Best regards,

Thomas Vial, Guillaume Marti and Julien Pompon.

In particular, please note the need to establish causative and not correlative links, in addition to providing much more mechanistic detail as to how DENV modulates AGPAT1.

Mosquito metabolomics is a nascent field that has the potential to shed new light on how DENV subdues its vector physiology. While our study provides an unprecedented description of the impact of DENV on the mosquito’s metabolome (multiple time points and tissue levels, and identification of mosquito-specific metabolites), our discovery that *AGPAT1* expression is regulated by DENV to modulate the lipidome and increase virus replication represents a significant mechanistic understanding with regards to the previous studies (i.e. Chotiwan et al, 2018. PLoS Pathogens), mostly based on a descriptive approach. Our study is the first to provide some insight on the mechanism by which DENV reconfigures the lipidome in mosquitoes. Indeed, AGPAT1 is the first-identified vector factor involved in DENV-induced metabolic reconfiguration. As such, we understand the interest of reviewers in obtaining more information on the mechanism: how is AGPAT1 regulated? How phospholipids influence DENV cycle? What stage(s) of DENV cycle is affected? … Although this is out of the scope of this paper, we now discuss the different hypotheses with regards to these interesting questions. We hope that the clear improvement in the understanding of DENV-induced metabolic reconfiguration we propose will be deemed a valuable incremental increase to our knowledge and worth publication to stimulate further research in the field of mosquito metabolomics.

Reviewer's Responses to Questions

**Part I - Summary**
Please use this section to discuss strengths/weaknesses of study, novelty/significance, general execution and scholarship.

Reviewer #1: The authors perform metabolomic analysis on dengue infected cells and mosquitoes, which broadly agrees with previously published metabolomic studies on dengue infection of mosquitoes. They find that dengue infection induces substantial changes to various aminophospholipid species. Further, they find the dengue infection substantially reduces the expression of AGPAT, a key enzyme in phospholipid synthesis. siRNA-mediated targeting of AGPAT moderately increases viral replication in cell culture and in mosquitoes. Further, they find that siRNA-mediated depletion of AGPAT phenocopies aspects of the metabolic changes induced by dengue infection. The identification of AGPAT1 as a restriction factor whose downregulation could be contributing to dengue-induced metabolic remodeling has implications for the field’s understanding of dengue-induced metabolic remodeling, but as indicated below the authors over-interpret a number of correlations implying causality, which, when coupled to a lack of mechanistic details of how dengue targets AGPAT1 or how AGPAT1 contributes to dengue infection limit the impact of the current work.

We thank the reviewer for acknowledging that our discovery of the first-identified DENV metabolic-related factor in mosquito has “implications for the field’s understanding of dengue-induced metabolic remodeling”.

To answer his comment about over-interpretation, we highlighted the limitations of our approach and discussed hypotheses about the mechanisms, as detailed below.

Reviewer #2: This study aims to understand how dengue virus (DENV) modulates mosquito metabolome to enhance virus production. Using high-resolution mass spectrometry, the authors identified changes in aminophospholipid (aminoPL) concentrations during the course of DENV infection in vitro and in vivo. They showed that the expression of 1-Acylglycerol-3-Phosphate O-Acyltransferase 1 (AGPAT1), a rate-limiting enzyme of phospholipid biogenesis, is decreased during DENV infection, whereas AGPAT1 knockdown leads to a modest increase in DENV production late in infection. This is a significant, but highly descriptive study. There is little mechanism as to how DENV utilizes a select group of phospholipids (PL) to enhance virus production. The study does not address how DENV infection causes AGPTA1 inhibition or the relationship between AGPAT1 inhibition and an increase in aminoPL concentration. It is unclear how in aminoPLs contribute to DENV infectivity in mosquitoes. Finally, if AGPTA1 plays a crucial role in DENV infectivity, its silencing should lead to a marked increase in DENV load. However, this is not the case in the current study.

We thank the reviewer for acknowledging the significance of our study. We also share his interests in obtaining a more detailed description of the mechanism. However, mosquito metabolomics is a new field that still lack the robust tools developed in the past decades in molecular biology. With the available tools and an in vivo approach (more relevant but harder to control for manipulative experiments), we propose here an incremental but significant improvement of how DENV subdues its vector physiology and an identification of metabolites influencing DENV. With regards to the modest effect of our metabolomic alteration, we want to emphasize that it is highly significant (p-value < 0.0012, 0.004 or < 0.0001), conserved in cells and at the mosquito levels, and that a previous artificial disruption of the metabolome in mosquito midguts also showed a moderate impact (Chotiwan et al 2018). A moderate impact may reflect the contributions of other factors not targeted in our reductionist approach.

Reviewer #3: In the manuscript entitled “Dengue virus inhibits AGPAT1 to alter phospholipids and enhance infection in Aedes aegypti,” Vial et al study the metabolome of Aedes Aegypti mosquitos over time after dengue infection. While other metabolomics studies of the mosquito midgut following Dengue infection have been published (Chotiwan, PLoS Pathogens 2018), this one is more comprehensive including Aedes Aegypti cells, midguts, and whole mosquitos. Furthermore, this study goes beyond the previous one by examining the role of a host lipid synthesis enzyme in this process. The strengths of this manuscript include the provision of a comprehensive database reflecting metabolic changes during dengue infection of mosquitos and the uncovering of a novel pathogenesis mechanism for Dengue in which a mosquito gene that inhibits viral replication is downregulated by the virus during infection. Weaknesses include the presentation of the findings and statistical analysis of the data.

We are very thankful to the reviewer for acknowledging that our study provides an improvement to the state-of-the-art both in the metabolic description and the identification of novel DENV host factor related to lipids. We fixed the weaknesses in presentation and statistics as indicated below.

**Part II – Major Issues: Key Experiments Required for Acceptance**
Please use this section to detail the key new experiments or modifications of existing experiments that should be absolutely required to validate study conclusions.

Generally, there should be no more than 3 such required experiments or major modifications for a "Major Revision" recommendation. If more than 3 experiments are necessary to validate the study conclusions, then you are encouraged to recommend "Reject".

**Reviewer #1:** Throughout the manuscript, the authors conclude that inhibition of AGPAT1 promotes infection by increasing aminophospholipids. While their data show a correlation between aminophospholipids levels, and dengue infection, a causal link between aminophospholipid levels and infection is never established. Are downstream aminophospholipid producing enzymes important for dengue infection? Does supplementation with specific aminophospholipids impact infection, e.g. in vitro?

We share the reviewer’s frustrations and questions but would like to argue that these limitations are inherent to the field of in vivo mosquito metabolomics. With available tools, we precisely (one enzyme depletion in a complex network) manipulated aminophospholipids and observed an impact on DENV.

1. Direct and controlled manipulation of aminophospholipids in vivo in mosquitoes is currently very challenging. Addition of specific aminophospholipids as suggested would not result in specific increase of the added phospholipids. The metabolic networks are highly dynamic and would reduce the supplemented compound to uncontrolled levels by transforming it into downstream products.

Instead, to alter the lipidome, we chose an indirect and somewhat more accurate method by reducing enzyme expression and measuring the impact on the lipidome.

Nonetheless, to clarify the limitations pointed out by the reviewer, and with which we agree, we now state in the first paragraph of the discussion:

Line 376 (from track-change version): “This suggests that DENV consumes/redirects aminoPLs, **although this is based on correlations between indirect lipidome alteration and virus multiplication**.”

2. Aminophospholipid producing enzymes could also be regulated by DENV and contribute to the DENV-induced metabolic reconfiguration. Although we chose to focus on an upstream enzyme and identified a DENV host factor, we hope that our work will stimulate further research on these downstream enzymes. We now clearly state that AGPAT1 depletion only partially reproduces the DENV-induced environment and that downstream aminophospholid producing enzymes may play a role:

Line 384 (track-change version): “That we only partially recapitulated the DENV-induced phospholipidome reconfiguration suggests that other enzymes, such as those directly producing and those hydrolyzing aminophospholipids [55], play a role.”

The authors claim, e.g. in the title, that dengue inhibits AGPAT1, this suggests inhibition of the protein. What the authors actually show is that AGPAT1 expression levels are down-regulated. What mediates this down regulation? Is live virus necessary for AGPAT1 down-regulation? Does UV-inactivated virus down-regulate AGPAT1 expression?

We modified the title and the text to clarify that we reported a gene expression change for *AGPAT1*.
The title now reads: “Dengue virus **reduces** ***AGPAT1*** **expression** to alter phospholipids and enhance infection in Aedes aegypti”. We clarified this as well throughout the text.

With regards to the mechanisms of *AGPAT1* expression alteration, we thank the reviewer for his suggestion and monitored *AGPAT1* expression upon infection with UV-inactivated DENV in cells (new Fig. S6). While panel Fig. S6A confirms the lack of replication for UV-inactivated DENV, Fig S6B showed no difference in *AGPAT1* expression between UV-inactivated DENV and mock infection at 24 and 48hpi.

This new mechanistic insight is now added to the results section:

Line 219 (from track-changes version): “To test whether virus replication was required for *AGPAT1* down-regulation, we incubated cells with UV-inactivated virus (Fig. S6A). AGAPT1 expression did not vary between mock and UV-inactivated DENV at 24 and 48hpi, indicating that active infection is required. “

Does AGPAT1 downregulation impact the phospholipid constituents of the virion? Or is the mosquito’s innate immune response to viral infection affected? In vitro, what aspects of the viral life cycle are impacted?

We thank the reviewer for raising these intriguing questions that emerge from our findings. Nonetheless, as mentioned above, we can only speculate about the answers in the discussion (fourth paragraph). DENV interacts with the membrane phospholipids at several steps of its cycle (entry, replication, translation, assembly and egress, as mentioned in the introduction and the discussion) and the change we report can potentially affect each of these.

To precise this point and highlight the reviewer’s hypotheses, we now state in the discussion that:

Line 410 (track change version): “DENV intricately interacts with aminoPL-containing membrane and a change as we report can alter several stages of its life-cycle, such as entry, replication, translation, assembly and egress.”

Further, the reviewer raises the hypothesis that immune response might be altered. To discuss this hypothesis, we now state:

Line 420: “Alternatively, *AGPAT* expressions could alter anti-viral immune response [58]. In mammalian cells, AGPAT overexpressions amplify cellular signaling of cytokine [62] that can reduce DENV infection in mosquito cells [61].”

Eventually, we conclude this paragraph by stating that: “Although the precise function of AGPAT1-regulated aminoPLs is unknown and may be multifactorial, previous studies and ours indicate that membrane aminoPL reconfiguration influences DENV multiplication”.

**Reviewer #2:** Fig. 1 addresses the impact of DENV infection on Ae. Aegypti metabolome. In Fig. 1B, the Venn diagrams and related triangles need to be explained.

To clarify the Venn diagrams, we modified the figure legend that now reads:

“(B) Venn diagrams show regulated metabolites at the different time points and bars indicate class distribution within tissue levels. Triangles indicate direction of regulation and are color-coded with regards to the time point.”

In addition, there are no controls for the heat maps in Fig. 1C, making it hard to judge the data.

We agree with the need to show control values and added a new heatmap figure in supplementary data (Fig. S4), while maintaining the original figure with fold changes. We think that displaying log2 fold change upon DENV infection relative to mock (as in Fig. 1C) simplifies the figure, facilitating interpretation of the data to reveal patterns. Fold change representation is widely used to represent metabolite changes across time course (see Chotiwan et al., PLoS Pathogens 2018, PMID: 29447265; Fontaine et al., Journal of Virology 2015, PMID: 25505078). The new Fig. S4 shows a heatmap with ion intensity in DENV and mock for each time point, showing control values as requested by the reviewer.

Fig. 2C-E show a decrease in AGPTA1 mRNA levels, during the course of DENV infection. These data need to be validated with AGPTA1 protein expression during DENV infection.

Although there is no commercially available antibody against mosquito AGPAT1, we tested an anti-human AGPAT2 polyclonal antibody (Novusbio, h00010555-d01p). We had defined that hAGPAT2 shares sequence similarity with AGPAT1 (Fig. 2B). Unfortunately, whereas the antibody detected AGPAT2 in human cell lysate (HuH7), it did not recognize any protein of the expected size (~30 kDa) in mosquito cell lysate (see figure below). Therefore, we can only measure gene expression, as it is commonly accepted for mosquito studies (e.g., Thomas et al., PLoS Pathogens 2018, PMID: 29494679; Olmo et al. Nature Microbiology 2018, PMID: 30374169).

**Figure. Immunoblot with anti-hAGPAT2 in Aag2 and Huh7 cell lysates.** 100µg of proteins were separated on SDS-PAGE and blotted with anti-hAGPAT2 polyclonal antibody. Beta actin level was used as loading control.

Fig. 3 addresses the impact of AGPAT silencing on aminoPL levels and DENV replication in mosquito cells. First, it will be helpful to show the control PL samples for the heat maps in Fig. 3C and 3E.

We added a new figure (Fig. S7) showing ion intensity for both DENV and mock conditions as requested. As proposed for Fig. 1C above, we kept the common visualization as fold change in the main figure to facilitate interpretation. However, we changed the format from heatmap to bar graphs with standard errors, as requested by reviewer 1 in part III. Following these changes, there are now two ways to present the data and indications of the variance, providing the readers with all information to interpret the results.

It is hard to compare the PL heat maps, from mock and DENV-infected cells, when they show different PL species.

Although regulated PL species differ in infected and mock conditions, we now facilitate interpretation of the phospholipid patterns by adding a figure panel with total number of phospholipids regulated upon mock and DENV infection (Fig. 3C). This will help compare DENV and mock as requested by the reviewer.

In Fig. 3D, it is also hard to interpret the DENV infection data based on the heat maps in Fig. 3C and 3E. There is only a modest increase in DENV production after AGPAT1 knockdown.

To better represent the change in DENV infection, we modified the scale from logarithmic to linear. The significant (p-value = 0.0012) increase of 2.1 fold upon AGPAT1 depletion is now clearly visible and should improve interpretation. Please see below for the new improved panel.

Fig. 4 addresses the impact of AGPAT1 silencing on DENV replication and aminoPL consumption in mosquitoes. Please, show the heat map for control siRNA-treated mosquitoes in Fig. 4B.

As for Fig. 1 and 3, we now present the ion intensity for both mock and DENV infection in a supplementary figure S9.

In Fig. 4CD, contrary to the authors assertion, AGPAT1 knockdown in mosquitoes shows no significant impact on DENV infectivity at day 2 or 7 postinfection. The DENV infection rate at day 7 (Fig. 4C) is clearly not impacted by AGPAT1 knockdown. There might be a slight increase in DENV genome replication at day 7, but is there a significant change in DENV load in these mosquitoes?

We are sorry if our previous statement was not clear. However, the data shows a significant (p-value = 0.004) 2.7 fold increase in DENV gRNA at 7dpi upon AGPAT1 depletion. We had also shown a significant increase in gRNA after infection with a lower inoculum (Fig. S10E). We transformed the y-axis scale from log to linear to better represent the changes.
Also, as requested to strengthen our findings, we now quantified infectious virus particles by plaque assay. In the new fig. 4D, we observe a significant (p-value < 0.0001) increase in pfu per infected mosquitoes at 7dpi upon AGPAT1 depletion (please see below).
Nonetheless, we acknowledge that infection rate and gRNA at 2 dpi were not affected. This is now clearly mentioned in the results section:
Line 342 (track-changes version): “However, the pro-viral impact of AGPAT1 depletion was not observed on infection rate (Fig. S10A) and at 2 dpi on gRNA (Fig S10B).”

**Reviewer #3:** 1) Figure 2: AGPAT1 expression is decreased in cells and whole mosquitos. Given that the virus disseminates from the midgut and that the decrease in AGPAT1 expression does not explain the observed increase in aminophospholipids in the midgut early in infection, the authors should also measure AGPAT1 and 2 expression in the mosquito midgut.

We thank the reviewer for the suggestion and now present data on *AGPAT1* and *AGPAT2* gene expression in midguts after mock and DENV infection (Fig. 2D). Similarly to cell and whole mosquitoes, we observed down-regulation of *AGPAT1* at 1 and 7 dpi, whereas *AGPAT2* expression was not modified.

2) Figure 3D: the control should have as many data points as the test samples.

The experiment was repeated to obtain the same number of biological replicates in each condition (Fig. 3F). These added samples did not change the previous pattern.

**Part III – Minor Issues: Editorial and Data Presentation Modifications**
Please use this section for editorial suggestions as well as relatively minor modifications of existing data that would enhance clarity.

**Reviewer #1**: A thorough metabolomics analysis of dengue infection in mosquitoes was previously published in Plos Pathogens in early 2018 (PMID: 29447265), which has somewhat similar findings. Similarities and potential differences should be addressed in the discussion.

We now added a comparison between the previous study and ours in the results section. We believe that this comparison in the results will not disturb the flow of our demonstration focused on our significant contribution: the discovery of AGPAT1 as a metabolic factor in mosquitoes.

The newly added comparison reads as:

Line 187 : “A large phospholipidome reconfiguration was previously observed in DENV-infected midguts at 3, 7 and 11 dpi [18]. However, in this previous kinetic study, PL were mostly up-regulated throughout the infection cycle. They reported a decrease in lysoPL abundance, which we observed at 14 dpi in mosquitoes. The authors also noted an increase in sphingolipids, which we saw in mosquitoes but only for one species. Those variations between the studies can stem from methodological differences in extraction and analyses, or from biological differences in mosquito colony and virus strain. Taken together, our results and those of others indicate that DENV infection reconfigures lipid membrane composition.”

In the abstract, the authors claim the dengue infection increases AGPAT1, when all their data indicates that dengue markedly reduces it. This is likely just a typo, but should clearly be fixed as it is a central conclusion.

We apologize for the typo and corrected the abstract that now reads:

“We showed that DENV infection **decreased** AGPAT1, but did not alter AGPAT2 expression in cells, **midguts** and mosquitoes.”

In Figure 1, its unclear which of the metabolites are significant. The figures would be easier to interpret with an added scheme to enable readers to quickly assess significance of specific changes.

All metabolites showed in Fig. 1 are significantly regulated upon DENV infection. To clarify this, we revised the caption that now reads:

“(C) Fold change of annotated and significantly regulated metabolites (|log_2_ fold change| > 1 and p-value < 0.05) by DENV in cells, midguts and mosquitoes as compared to mock infection.”

Additionally, we provided a scheme of our metabolomics workflow in Fig. S2, which is now referenced in Fig. 1 caption.

In Figure 3C and 3E, the data should not be shown as a heat map. They should be plotted so that fold changes and data variance/errors can be easily assessed.

We agree that Fig 3C and 3E are clearer as bar graph and plotted them as such (now renumbered Fig 3D and 3F). For clarity and consistency, we also plotted similar data in Fig 4E and 4F.

**Reviewer #2:** The manuscript needs editing.

We apologize for editing errors and have paid particular attention to it in the new version, plus we had it reviewed by a professional scientific editor.

**Reviewer #3:** 1) Abstract: While the abstract length is limited, it is critical to spell out AGPAT and its function in one sentence so readers are not left in the dark. An indication of which aminophospholipids the study is focused on might also be helpful.

We revised the abstract accordingly by adding:

1. Information about AGPAT function

“We phylogenetically characterized **acylglycerolphosphate acyltransferase** (AGPAT) enzyme isoforms to identify those that catalyze a rate-limiting step in phospholipid biogenesis, **the acylation of lysophosphatidate to phosphatidate**.”

2. The classes of aminophospholipid on which the study is focused:

“Our study suggests that DENV regulates aminophospholipids, **especially phosphatidylcholine and phosphatidylethanolamine,** by inhibiting AGPAT1 to increase their availability for virus multiplication.”

2) Line 22: Is this a typographical error? Figure 2 shows that Dengue infection decreases expression of AGPAT1 rather than the opposite.

We apologize for the typo and corrected the abstract:

“We showed that DENV infection **decreased** AGPAT1, but did not alter AGPAT2 expression in cells, **midguts** and mosquitoes.”

3) Line 39, sentence beginning with “however:” This sentence is confusing and should be rewritten.

We corrected the sentence as:

Line 42 (track-changes version): “However, occurrence of insecticide resistance diminishes vector control efficacy.”

4) Line 147: “PL were the most regulated.” This is a vague statement. Do the authors mean that the largest changes in abundance were seen for these metabolites over the course of the infection?

To clarify our observation, we modified the sentence that now reads:

Line 154 (track-changes version): “The PL class had the highest number of regulated metabolites in the midgut and mosquito…”

5) Lines 151-189: In this section, the authors describe in detail the changes in different types of lipids without adding any insight into processes that are at work. These changes are already apparent in Figure 1 and could also be gleaned from supplementary datasets. I would suggest shortening this section and only describing in prose changes that provide insight into the possible underlying mechanisms.

We shortened this section of the results (Line 158 to 184), removing details on metabolite species that can be observed in the figure, as requested.

6) Lines 194-5: Please describe what “the reaction” is.

The reaction is now detailed in the text:

Line 201 (track-changes version): “Motifs I and IV bind to acyl-CoA and catalyze **the lysoPA to PA acylation**…”.

7) Lines 208-9 and Figure 2: Upregulation of AGPAT2 is not convincing and not significant. I would recommend leaving this statement out.

We modified the text accordingly:

Line 216 (track-changes version): “In contrast, AGPAT2 was not significantly regulated in cells, midgut and mosquitoes (Fig. 2C-E).”

And the Figure 2 title to remove observation that AGPAT2 was regulated:
“**Figure 2. DENV infection decreases *AGPAT1* but not *AGPAT2* expression.”**

8) Figure 2: AGPAT1 expression is decreased in cells and whole mosquitos. Given that the virus disseminates from the midgut and that the decrease in AGPAT1 expression does not explain the observed increase in aminophospholipids in the midgut early in infection, the authors should also measure AGPAT1 and 2 expression in the mosquito midgut.

We quantified AGPAT1 and 2 in midguts at 1 and 7 dpi and included this new data in Fig. 2D. We observed the same trend in midguts as in cells and mosquitoes: DENV decreases *AGPAT1* but not *AGPAT2* expression.

9) Figure 3D: The increase in PFU is quite small, suggesting that this may be a minor contributor. More importantly:
An unpaired t-test is used to assess statistical significance. A multiple comparisons test such as an ANOVA would be a more appropriate statistical test to apply here.

We used an ANOVA followed by Dunnett’s post hoc test to test the impact of AGPAT1 depletion and obtained a significant p-value (0.0012).
While the impact of AGPAT1 depletion on viral titer may appear modest, it is significant and to better represent the change in pfu/ml (2.1 fold increase), we changed the y-axis scale from logarithmic to linear in renumbered Fig. 3E (see below).

10) Methods: The authors state that they used an unpaired t-test to analyze the metabolomics data. The authors should specify which condition was used as the “control.” In addition, for metabolomics time courses such as that shown in Figure 1C, a multiple comparisons test should be used to assess statistical significance rather than an unpaired t test.

Physiological and metabolic states of each time-tissue combination may greatly vary and neutralize part of the DENV-induced changes. For instance, at 1 dpi the blood is not digested and its composition influences midgut and mosquito metabolome. Evidently, there are also great metabolic differences between cells and whole mosquitoes. To normalize these, we compared infected to uninfected within each time-tissue combination and used an unpaired t-test.

To clarify this, we modified the methods section:

Line 516 (track-changes version): “To account for physiological variations between the different time-tissue combinations, the t-tests were done by comparing the same tissue in infected and uninfected conditions within each time. Uninfected condition was used as control.”

11) Methods: Please specify when replicates are experimental and when they are technical replicates rather than describing as “repeats.”

In methods and figure captions, we changed the term “repeats” for “biological replicates”.
