# Supplementary material for: Dengue virus reduces AGPAT1 expression to alter phospholipids and enhance infection in Aedes aegypti
Source: PLoS Pathog. 2019 Dec 9;15(12):e1008199. doi: 10.1371/journal.ppat.1008199 (PMC6922471; doi:10.1371/journal.ppat.1008199)
Supplement: Attachment — Submitted filename: PPATHOGENS-D-19-00992R1 answer to reviewers comments.docx [file ppat.1008199.s024.docx]

**PPATHOGENS-D-19-00992R1**

Dear Editors,

As requested, we generated new data to test whether phospholipid supplementation can alter DENV multiplication in mosquito cells. In support of our hypothesis, ethanolamine supplementation that alters phosphoethanolamine composition rescued the AGPAT1-depletion phenotype.

Please find below a point-by-point answer to the comment in blue.

Best regards,

Thomas Vial, Guillaume Marti and Julien Pompon.

The editors have reviewed your revision and would like you to experimentally address some of the points made by Reviewer #1.

In particular, is the issue of whether phosphatidic acid (or its downstream products) is inhibitory to DENV replication in vitro (Aag cells) both in wild type infection and the silencing experiments. We view this as important for two reasons.

(i) It further controls the silencing experiment for off-target effects and directly implicates the lipid pathway.

(ii) It asks the important question as to what happens when DENV can't downregulate phosphatidic acid. Silencing AGPAT1 likely produces a minor 2-fold phenotype on DENV replication because DENV infection is already decreasing its expression level.

This experiment can be done via simple media addition of PA over a range of concentrations or over-expression of AGPAT1 if necessary. qRT-PCR and titering would give some insight into life cycle stage (controlling for cell viability). This should be a relatively simple experiment. An additional in vivo experiment is unnecessary, as it is difficult to adequately control.

We first supplemented the media with phosphatidic acid (PA). However, supplementation of media with 20 or 2mM of PA was toxic to the cells within 24h (all cells detached).

Second, we supplemented the media with 2mM of ethanolamine. PA produces diacylglycerol (DAG), to which an ethanolamine group is added to form phosphoethanolamines (PE), one of the phospholipids regulated by AGPAT1 depletion. Addition of ethanolamine, the PE precursor, modified the phospholipid composition based on literature (Lipton et al., JBC, 1990). We thus supplemented the media with a precursor of PE. We observed that ethanolamine supplementation reverted the DENV gRNA increased induced by AGPAT1 depletion.

These results are added to the result section l. 226-238 from the final version and in fig. S9.

This added figure (i) confirms the role of lipid in AGPAT1 increase of DENV multiplication and (ii) reveal the importance PA downstream products.
